# Enhanced forecasting of bird nocturnal migration intensity in relation to previous days and synoptic weather patterns

**DOI:** 10.1007/s00484-025-02917-4

**Published:** 2025-04-19

**Authors:** Amédée Roy, Thibault Désert, Vincent Delcourt, Cécile Bon, Baptiste Schmid

**Affiliations:** 1 France Energies Marines, 525 Avenue Alexis de Rochon, Plouzané, 29280 France; 2 Météo-France, 42 Avenue Gaspard Coriolis, Toulouse, 31100 France; 3 Biotope, 22 Boulevard Maréchal Foch, Mèze, 34140 France; 4 Swiss Ornithological Institute, Seerose, 1, Sempach, CH-6204 Switzerland

**Keywords:** Bird migration, Weather radars, Aeroecology, Forecast, Radar monitoring, Weather dynamics, Synoptic weather, Functional principal component analysis, Empirical orthogonal functions, Shapley values

## Abstract

Operational bird migration forecast models have recently offered promising perspectives for mitigating the impacts of human activities on avifauna. These models improve on simple phenological expectations by harnessing the intricate relationship between bird movements and weather conditions to forecast migration fluxes days in advance. However, state-of-the-art models face limitations as bird fluxes are often simply modelled as a response to local and instantaneous weather without accounting for previous and synoptic weather patterns. This study focuses on enhancing bird migration forecasts by evaluating the contributions of weather dynamics at various spatial and temporal scales. We use bird vertical density data from 9 French weather radars over 6 years and employ gradient-boosted regression trees for predictions. Dimension reduction tools are used to describe local and continental-scale weather conditions from the previous three days. We also explore the contributions of the different meteorological metrics considered using explainable regression trees tools. Our model improved phenology models by explaining about 1.3 and 2.25 times more additional variance than approaches based on local and instantaneous weather conditions in spring and autumn, respectively. Local and instantaneous weather metrics contributed the most, but they mainly helped identifying nights with low migration. In contrast, weather metrics for previous 3 days were crucial to forecast highest intensity migration events, as they enabled to account for bird accumulation in relation to unfavorable weather locally and remotely. This study enhanced forecast accuracy and contributed to a deeper understanding of the factors influencing bird migration. It enabled the identification local and synoptic weather patterns related to important migration events without a priori knowledge. It is therefore easy to interpret, easy to transfer to other ecological systems, and promising for the accurate forecast of migration peaks. Forecasted peaks can guide conservation efforts, for example by dimming lights for birds at night or by shutting down wind turbines.

**Supplementary Information:**

The online version contains supplementary material available at 10.1007/s00484-025-02917-4.

## Significance statement

Nocturnally migrating birds undergo large distances and their motivation to migrate is linked to weather conditions. We here relate the local, synoptic (i.e. continental scale), and preceding weather conditions to forecast peaks and lows in migration intensity. This methodological framework enhanced accuracy of our forecasts and deepened our understanding of the underlying ecological process. It is particularly valuable for the design of relevant bird migration forecast tools to mitigate the impacts of human activities on avifauna.

**Supplementary Information:**

The online version contains supplementary material available at 10.1007/s00484-025-02917-4.

## Introduction

Billions of birds migrate worldwide, traveling seasonally to optimal habitats (Dokter et al. 2018; Hahn et al. 2009; Nussbaumer et al. 2021a). Migration is a fundamental part of their life cycle, important for both survival and reproduction (Newton 2008). However, bird migration is associated with various risks, including natural ones such as predation, extreme weather, and starvation (Newton 2007), as well as human-induced risks, including habitat loss, collisions with vertical structures, hunting or disturbance due to artificial light at night (Hüppop et al. 2006; Jiguet et al. 2019; La Sorte et al. 2017; Rigal et al. 2023). With recent increases in light pollution and in the utilization of airspace due to buildings, power lines, aircrafts, or wind turbines, there is an urgent need to mitigate these human-induced impacts (Bauer et al. 2017). Forecast models of temporal patterns of migration are often seen as critical tools for real-time management and planning of human activities (e.g. for artificial lighting reduction and wind turbine shutdowns). For this reason, operational models have recently been developed to forecast nocturnal bird migration fluxes up to three days in advance at specific locations (Bradarić et al. 2024; Kranstauber et al. 2022; Van Belle et al. 2007) and at a continental scale (Van Doren and Horton 2018).

The seasonal phenology of bird migration, defined as the interannual average of the migration intensity, is relatively well-known. The real challenge in forecasting bird migration lies in the fluctuations around this phenology. Consequently, the key concept of operational forecast models is to learn the associations between these fluctuations and weather conditions. These relationships, when applied to numerical weather forecasts that span several days ahead, allow to forecast the associated bird migration fluxes for the same forecast period. The responses of migratory birds to weather conditions are however intricate and depend on a multitude of weather factors that influence bird behavior across multiple spatio-temporal scales (Becciu et al. 2019; Shamoun-Baranes et al. 2017). Early work demonstrated the link between bird migration departure and synoptic (i.e., large scale) weather systems (Lack 1960; Richardson 1978, 1990). Migratory bird departures are often associated with anticyclonic systems characterized by high atmospheric pressure, with generally low winds, dry weather, and clear skies (Clipp et al. 2020; Cooper et al. 2023; Manola et al. 2020). In addition, migration strategies also vary depending on the conditions birds encounter locally during their migration. Indeed, several studies mention the fact that birds can adapt their flight altitudes to reach supportive winds, thus reducing the energy to be expended for a given distance to travel (Kemp et al. 2013; Schmaljohann et al. 2009). In addition, the migration of many individuals is often interrupted by rain and poor visibility (Erni et al. 2002; Kranstauber et al. 2022; Van Belle et al. 2007; Van Doren and Horton 2018). Temporal changes in weather have also a strong influence on the decision-making of migrating birds, including migration departure (Cooper et al. 2023; Erni et al. 2002; Kranstauber et al. 2022). For example, prolonged periods of poor weather conditions lead sometimes to an accumulation of migrants waiting for better conditions (Kranstauber et al. 2022; Shamoun-Baranes et al. 2017). This has been notably observed in front of some geographical barriers such as large water bodies, after several days of strong headwinds and/or precipitation (Clipp et al. 2020).

To model and forecast bird responses to weather, existing approaches have recently benefitted from data from weather radars networks for describing the fluxes of nocturnal bird migration. Weather radars are a promising tool for bird migration monitoring as they provide quantification of the biomass movement across continent-wide scales (Bauer et al. 2017; Shamoun-Baranes et al. 2014). Various statistical regression tools have been employed to predict bird vertical density profiles (or aggregated density) derived from weather radars. They include generalized linear models (Erni et al. 2002; Van Belle et al. 2007), generalized additive models (Kranstauber et al. 2022), regression trees (Van Doren and Horton 2018), and more recently, deep neural networks (Lippert et al. 2022; Mao et al. 2023). These statistical models have shown promising results, however, they still face limitations when it comes to predicting events of highest migration densities, referred to as ‘peaks’ (Kranstauber et al. 2022; Van Doren and Horton 2018). Moreover, they have primarily focused on local and instantaneous weather data at the time and position of the predicted bird density. Some studies have tested the influence of the previous night’s local conditions, but they often neglect considering synoptic weather dynamics (Erni et al. 2002; Kranstauber et al. 2022; Van Doren and Horton 2018). Many studies suggest yet that weather dynamics at a continental scale might also contribute significantly to bird migration forecasts (Clipp et al. 2020; Manola et al. 2020). Finally, many studies have focused on predicting vertically integrated bird flows, thus ignoring vertical distributions which are nevertheless crucial for real-time management and conservation strategies.

In this study, we address the problem of forecasting vertical profiles of bird densities, derived from weather radar data, using numerical weather forecasts. Our main contribution is in the characterization and quantification of the contribution of local weather conditions as well as synoptic weather patterns from previous days on forecast models. We also offer several recommendations for forecasting such a complex ecological process and discuss potential areas for improving the prediction of migration fluxes, including peaks.

## Material & methods

### Data formatting

#### Bird density

Bird densities were estimated using 9 dual-polarized weather radars from the French Meteorological Institute: Abbeville, Bordeaux, Bourges, Falaise, Nancy, Nîmes, St-Nizier, Toulouse and Treillières (see Supplementary Materials). We used the ‘vol2bird algorithm (Dokter et al. 2019) for computing averaged bird density profiles. It was parameterized with a correlation coefficient threshold of 0.95 and a fringe size of 45km, based on a preliminary study conducted over a week of migration interrupted by precipitation events. The goal was to overcome the contamination of bird density estimation by precipitation at the fringes of large cells and within small cells. This guarantees low contamination but might result in the loss of a small part of the biological signal during rainy events. The estimated bird density in individuals per km^3^ (ind.km^− 3^) was calculated from the volumetric reflectivity based on an average Radar Cross Section of 11 cm^2^ following (Dokter et al. 2011). We selected a dataset including 9 defined time-points for deriving nightly vertical profiles: 20:00, 20:15, 20:30, 23:00, 23:15, 23:30, 02:00, 02:15, and 02:30 UTC, representing early night, middle of night, and late night. We selected all nights from February to May and from July to November for 6 consecutive years (2017–2022). To address potential misestimations of bird intensity vertical profiles related to weather radar data processing, we averaged the profiles hourly (at h + 0, h + 15 and h + 30 min) to obtain three density profiles per night and per radar. We removed incomplete vertical profiles that had bird intensity estimations for fewer than five altitude bins per profile. The profiles were then linearly interpolated on a vertical scale from 0 to 3000 meters above ground level in steps of 250 meters to match numerical weather forecast vertical axis. We excluded insect-dominated events using a Gaussian mixture model as presented in Nussbaumer et al. (2021) (Details given in Supplementary Materials). We finally log-transformed bird densities to reduce the skewness of bird density distributions. For the sake of simplicity, when we mention “bird densities” in this manuscript, please note that we are referring to bird densities that have undergone a logarithmic transformation.

### Weather data

We compiled data from two numerical weather models developed by Météo-France, AROME and ARPEGE. With a high spatiotemporal resolution (1.3 km, 1 h), the AROME model is particularly relevant for describing local scale weather conditions within France (Seity et al. 2011). We extracted hourly data from AROME reanalysis products at each radar location during all nights (18:00 to 06:00 UTC) from 2017 to 2022. We selected zonal (i.e., eastward) and meridional (i.e., northward) wind speed components (m.s^− 1^), atmospheric pressure (Pa), temperature (K), and humidity (%) at 13 different height levels from 0 to 3000 m above ground by 250 m steps. We also extracted lower-level cloudiness (%) and accumulated precipitation on the ground over the last hour (kg.m^− 2^). In addition, we used ARPEGE, a global model that describes synoptic weather patterns. We computed nightly average of sea surface atmospheric pressure (Pa), 850 hPa isobar temperature (K), 850 hPa isobar humidity (%), and lower-level cloudiness (%) from reanalysis products. These metrics were computed for every night from 2017 to 2022, within the longitude range of -20° to 10° and the latitude range of 10° to 70° and using 0.5° mesh grids.

It is important to note that we only used weather data derived from reanalysis products (i.e. numerical forecasts corrected a posteriori with in-situ measurements). This is relevant to better identify and quantify the previous and synoptic weather patterns related to bird migration with high quality weather data. However, the predictive performance metrics presented in this study consequently underestimate operational forecast errors, as we do not account for the error of numerical weather forecast model with increasing lead-time.

### Dimensionality reduction

To reduce the dimensionality of bird fluxes and weather data, we employed different data transformations. We applied Functional Principal Component Analysis (FPCA) to the vertical profiles of bird density, wind, humidity, temperature, and atmospheric pressure. FPCA enables the identification of the main patterns of variability in vertical profiles, which are referred to as principal components. For each metric, we selected two distinct principal components. Each principal component is associated with a weight, which describes its importance to the overall variation in the data over time (Ramsay and Dalzell 1991), noted hereafter PC_FPCA_.

Furthermore, we transformed the synoptic weather patterns from ARPEGE using Empirical Orthogonal Functions (EOF). Similar to FPCA, EOF decompose a spatiotemporal process into a few principal components of spatial variation, each associated with a weight that indicates their importance over time (Hannachi et al. 2007). In this case, we used four distinct principal components for each metric, which allowed us to describe large-scale maps of weather metrics with few parameters, noted hereafter PC_EOF_.

#### Removing climatological trends

Since we aimed to forecast the fluctuations around migration phenology, we removed the seasonality in bird density time series. This was done by computing the standardized anomalies of the two PC_FPCA_ of bird density profiles. Standardized anomalies are calculated by dividing the anomalies of PC_FPCA_ from the climatological mean by the climatological standard deviation. Similarly, we removed climatological trends for all weather metrics. More precisely, we computed anomalies of PC_FPCA_ of wind, pressure, temperature, and humidity profiles, as well as anomalies of lower-level cloudiness and accumulated precipitation on the ground. Anomalies were also computed for large-scale weather data before the computation of PC_EOF_.

### Gradient-boosted regression trees

#### Training & validation

We used gradient-boosted trees, a machine learning technique, to forecast bird migration density from weather data. We relied on the implementation of LightGBM known for its efficiency with large amounts of data (Ke et al. 2017). Following (Kranstauber et al. 2022), we built separate models for spring and autumn migrations. Each model was trained and validated using cross-validation over several years. In other words, for a specific tree configuration, we trained the model over five of the six available years and then evaluated its performance over the remaining year. By repeating this process for the six years of the dataset, we estimated the performance of a model by calculating the average R^2^ score. The architecture (number of trees, leaves per tree, etc.) and training hyperparameters (loss function, learning rate, regularization terms, etc.) of each model were optimized on this score using a dedicated optimization algorithm, namely the Tree-structured Parzen Estimator (Bergstra et al. 2011). Domains of considered hyperparameters are given in Supplementary Materials.

#### Forecasting the standardized anomalies of the PCs of bird density profiles

We trained models to predict standardized anomalies of the two PC_FCPA_ of vertical bird density profiles, for the two seasons separately. These models took as input 117 explanatory variables, as follows:

- Radars’ characteristics: longitude, latitude, and antenna height above sea level.
- Time characteristics: solar declination as a proxy for day of year (apparent position of the Sun in the sky; yearly periodicity), and angular transformation of time of day (rotation of the Earth relative to fixed stars; daily periodicity).
- Anomalies of the two PC_FCPA_ of zonal wind, meridional wind, pressure, temperature, and humidity profiles at the forecasted time and nightly averaged for each of the last 3 nights.
- Anomalies of cloudiness and precipitation at the forecasted time and nightly averaged for each of the last 3 nights.
- The four PC_EOF_ of pressure, temperature, humidity, and cloudiness anomalies at the forecasted time for each of the last 3 nights.

#### Contributions of input features

We identified the contributions of the different predictors for model predictions using Shapley additive explanations (SHAP). SHAP values, based on the game-theoretic Shapley value, facilitate the quantification of input contributions for individual predictions (Lundberg et al. 2020). Absolute SHAP values indicate the strength of a feature’s impact on the model’s output, with positive and negative values indicating an increase or decrease of the response variable, respectively. SHAP values can also be added together to estimate the combined contribution of a group of features, providing a global interpretation over regression trees. In this study, we estimated the average contribution of different groups of input features:

- The contributions of “Eastward wind”, “Northward wind”, “Temperature”, “Pressure”, “Humidity”, “Cloudiness”, and “Precipitation” were evaluated by adding SHAP values for all features derived from each of these weather metrics.
- The contributions of “Local” and “Synoptic” weather metrics were evaluated by adding SHAP values for all features defined locally or at the synoptic scale at the time of the prediction.
- The contributions of “Local previous 3 days” and “Synoptic previous 3 days” weather metrics were evaluated by adding SHAP values for all features defined locally and at the synoptic scale for the previous 3 days.

#### Characterization of favorable and unfavorable weather patterns

SHAP values were also used to characterize the favorability of distinct weather patterns. We defined “favorable” and “unfavorable” conditions as input features with positive and negative SHAP values, respectively. By averaging input of features associated with favorable conditions, we were able to reconstruct favorable weather vertical profiles and synoptic weather maps from averaged PC_FPCA_ and PC_EOF_. This enabled insights on the specific weather temporal patterns identified and used by the gradient-boosted trees to predict bird migration events.

#### Benchmark models

To evaluate the benefits of our approach we compared our framework with other approaches to bird migration forecasts. First, we considered a naïve approach simply based on the phenology trend. Without including any weather data, this approach provides a way to forecast bird migration based on the seasonally-averaged observed densities. In addition, we trained gradient-boosted trees to predict bird densities for each altitude of density profiles, similarly to (Van Doren and Horton 2018). To this end, we included the height above ground as supplementary input feature and considered only local and instantaneous raw weather metrics at the specific height. We also trained gradient-boosted trees very similar to our model, using PC_FPCA_ of the vertical bird density profile as target features and including solely local and instantaneous weather metrics (i.e. without the metrics related to synoptic weather and previous days conditions).

All gradient-boosted trees were trained using the same training and validation datasets, as well as the same hyper-parameter tuning framework. Hereafter, we refer to the Van Doren and Horton (2018) framework, as the forecast modelling framework that we have applied to our dataset.

#### Evaluation metrics

To be able to compare the performance of distinct forecasts model, it is crucial to compute predictive scores on the same metrics. To this end, the standardized anomalies of vertical bird density PCs were transformed back into profiles of predicted bird density (in log-transformed ind.km^− 3^.). We evaluated overall scores using the cross-validated average R^2^ score. We also evaluated the ability of a model to identify main migration peaks using a metric from binary classification tasks, the F1-score. We identified the peaks at each radar location, which we defined as the top 5% (0.95 quantile) of the highest bird density values.

## Results

### Phenology trend

**Fig. 1:**
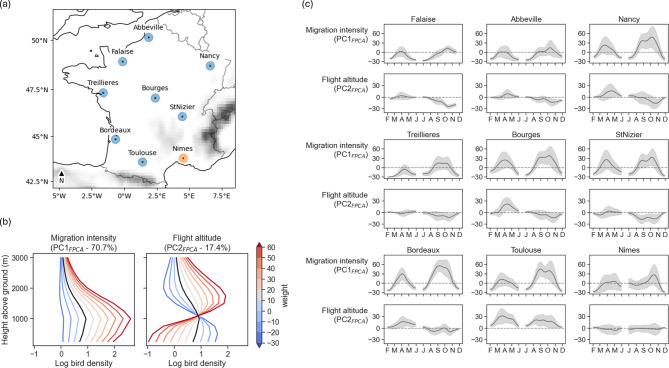
(**a**) Location of the nine French weather radars considered in this study (blue and orange circles refer to C- and S-band radars respectively). Greyscale background reflects topography. (**b**) The two main components of the functional principal component analysis of the vertical profiles of log-transformed bird densities: PC1_FPCA_ depicts variations in migration intensity, PC2_FPCA_ variations in flight altitude. The black profiles refer to the averaged density profiles over locations and time. Colored profiles describe the variation of log bird density profiles for different values of PC_FPCA_. (**c**) Phenology (i.e. smoothed daily average) of the main components of bird density profiles at each radar location. Envelopes refer to smoothed daily standard deviations

Dimension reduction by Functional Principal Component Analysis (FPCA) of the vertical profile of log-transformed bird densities resulted in two primary components (88% variance explained). The first component (PC1_FPCA_, 70.7%) was positively associated with the migration intensity and the second component (PC2_FPCA_, 17.4%) was positively associated with the average flight altitude (Fig. 1b). The highest migration intensities (PC1_FPCA_) were observed in a main migration corridor crossing France from the north-east to the south-west (refer to Fig. 1c). Migration intensity (PC1_FPCA_) was generally higher in autumn compared to spring across all radar locations. Conversely, flight altitudes (PC2_FPCA_) tended to be higher in spring and were particularly high in Toulouse.

### Bird migration forecast performance

**Fig. 2:**
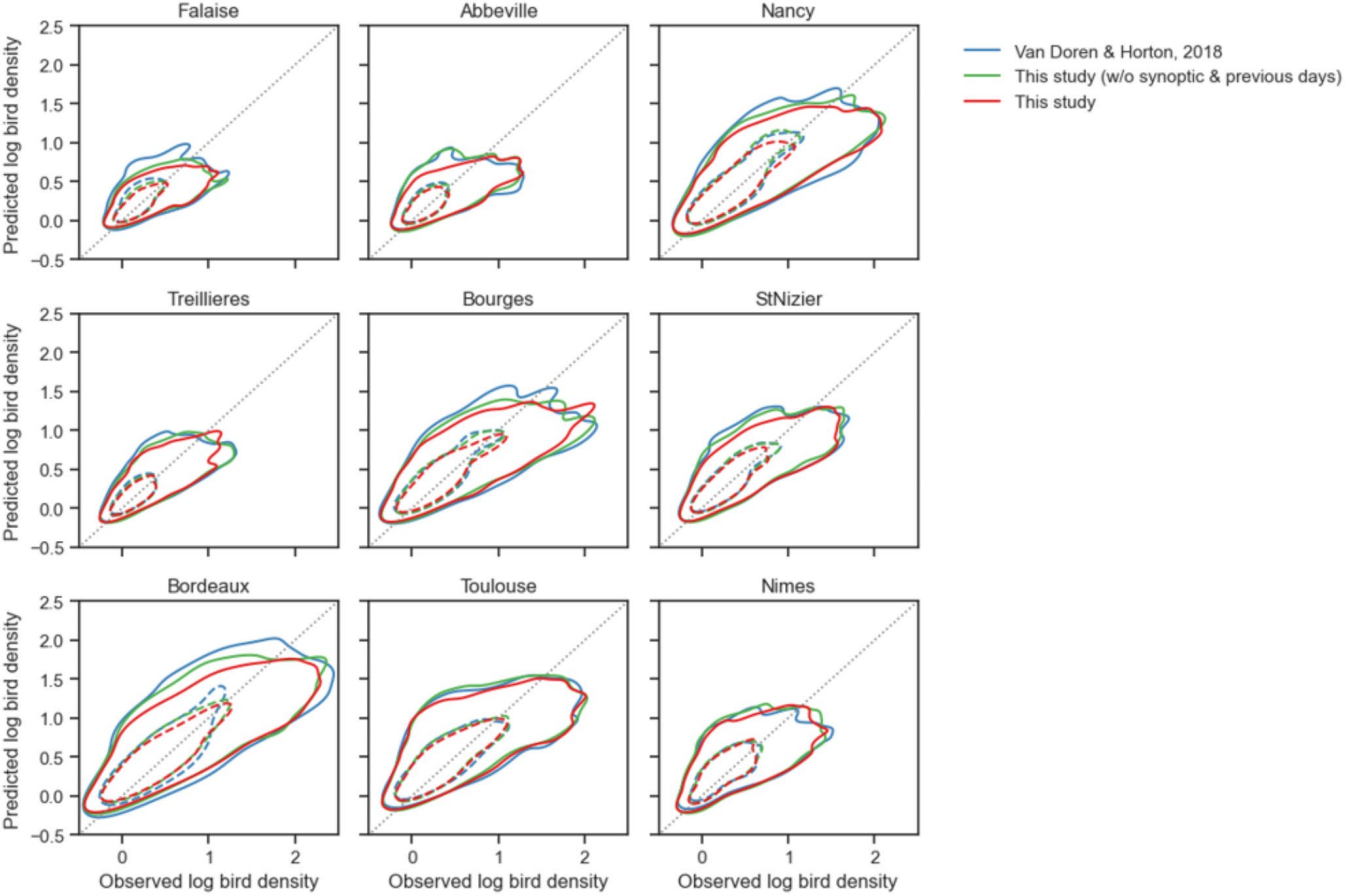
Predicted vs. observed migration log bird density for the three predictive models. Line and dashed envelopes show 50% and 10% iso-proportions of the density. Dotted gray line indicates unity

**Fig. 3:**
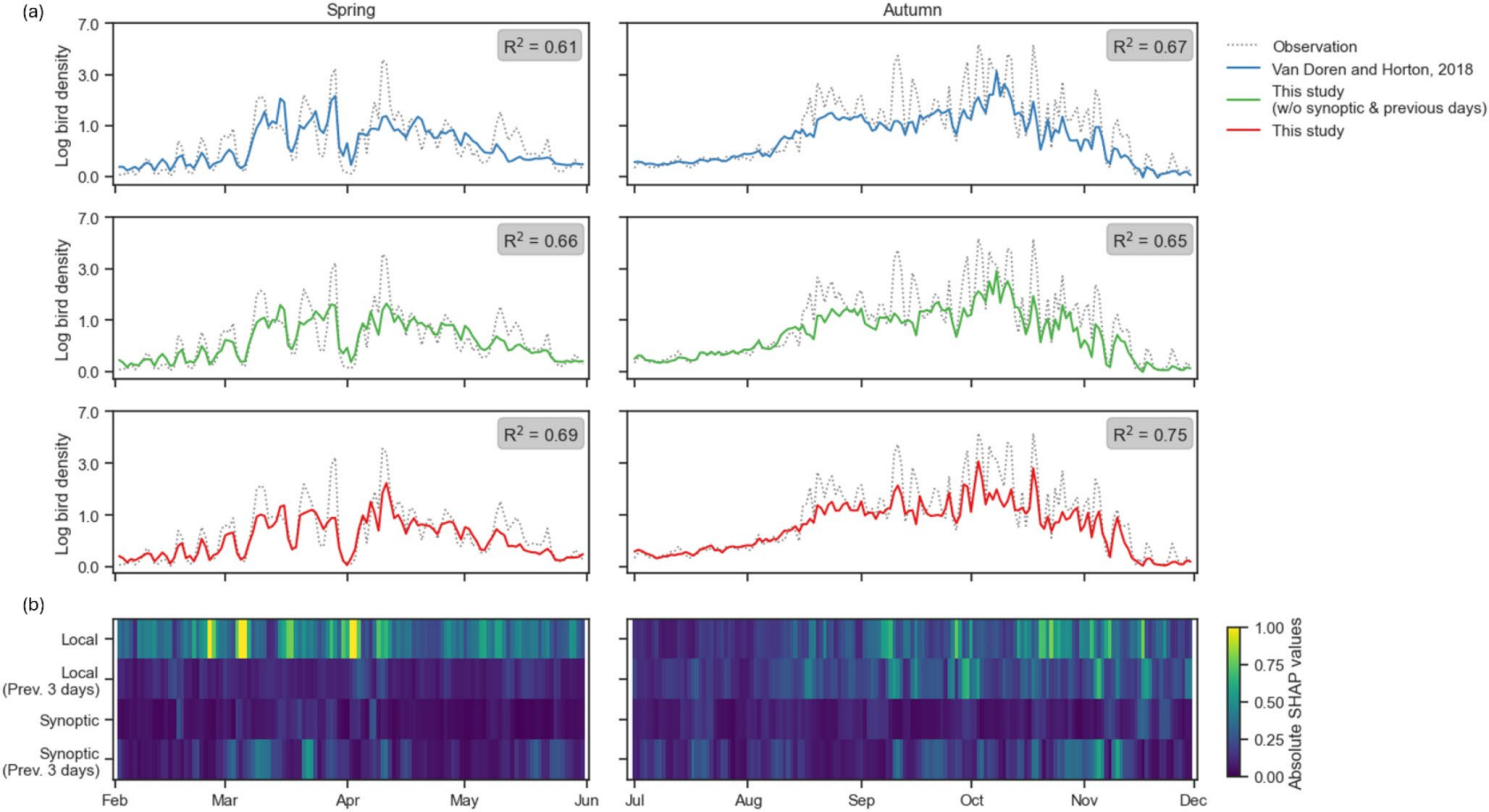
(**a**) Averaged bird density predictions for year 2022 given by three predictive models. (**b**) Color matrixes refer to the contributions of explanatory covariates aggregated in four groups through absolute SHAP values: Local and instantaneous weather metrics (Local), local and 3-day temporal weather metrics (Local Prev. 3 Days), synoptic and instantaneous weather metrics (Synoptic), and synoptic and 3-day temporal weather metrics (Synoptic Prev. 3 Days). Left and right panels refer to respectively spring and autumn migrations

Our approach, which employs gradient-boosted trees and dimension reduction tools, has consistently demonstrated superior predictive accuracy over benchmark models for all years and seasons, except for spring 2020. This approach accounted for local and synoptic weather temporal patterns and explained approximately 47% and 60% of the variance in bird densities during spring and autumn, respectively (Table 1). Interestingly, the naïve forecast based on phenology trends explained a substantial part of variance (35% and 51%, in spring and autumn respectively) (Table 1). The inclusion of local and instantaneous weather data, as per the methodology proposed by Van Doren and Horton (2018), resulted in an increase in the explained variance by 9% and 4% from the phenology model. In comparison, our model accounting for previous days and synoptic weather patterns resulted in an increase in the explained variance by 12% and 10% in spring and autumn, respectively. Therefore, our model explains about 1.3 times (for spring) and 2.25 times (for autumn) more the part of variance that is not related to phenology, compared to the approach by Van Doren and Horton (2018). Approaches that did not incorporate weather information for previous days tended to overestimate more often bird densities, particularly in the main migration corridor and in the north-west part of France (see Fig. 2). As a result, our best approach significantly improved the identification of nights with the highest migration intensity, as evaluated using the F1-score in both spring and autumn (Table 1). Despite these improvements, it still underestimates substantially the highest density values (Fig. 3).

**Table 1:** Performance of benchmarked models as well as this study’s model by year and season. The overall score is a R^2^-score used to evaluate the percentage of explained variance by each model. The peak score is based on a classification performance metric, the F1-score, evaluating the ability of the different models to identify migration peaks

		Overall (*R*^2^ score)				Peaks (F1 score)			
		*Phenological trend*	Van Doren and Horton (2018)	*This study* *(w/o synoptic & previous days)*	*This study*	*Phenological trend*	Van Doren and Horton (2018)	*This study* *(w/o synoptic & previous days)*	*This study*
Spring	2017	0.391	0.498	0.512	0.528	0.388	0.493	0.537	0.537
	2018	0.355	0.377	0.406	0.411	0.409	0.477	0.500	0.455
	2019	0.366	0.510	0.532	0.533	0.246	0.362	0.377	0.464
	2020	0.333	0.400	0.451	0.437	0.218	0.276	0.379	0.425
	2021	0.332	0.471	0.481	0.488	0.179	0.436	0.423	0.500
	2022	0.334	0.384	0.430	0.441	0.237	0.355	0.376	0.419
	**Total**	**0.35 ± 0.024**	**0.44 ± 0.06**	**0.47 ± 0.049**	**0.47 ± 0.051**	**0.28 ± 0.095**	**0.4 ± 0.083**	**0.432 ± 0.07**	**0.47 ± 0.045**
Autumn	2017	0.545	0.593	0.594	0.640	0.483	0.467	0.533	0.583
	2018	0.533	0.477	0.513	0.635	0.418	0.377	0.328	0.574
	2019	0.463	0.531	0.561	0.583	0.243	0.262	0.340	0.408
	2020	0.491	0.552	0.560	0.569	0.310	0.353	0.293	0.362
	2021	0.523	0.587	0.586	0.593	0.396	0.333	0.288	0.459
	2022	0.532	0.576	0.577	0.609	0.323	0.406	0.436	0.481
	**Total**	**0.51 ± 0.031**	**0.55 ± 0.044**	**0.57 ± 0.029**	**0.61 ± 0.029**	**0.36 ± 0.087**	**0.37 ± 0.069**	**0.37 ± 0.096**	**0.48 ± 0.088**

### Contributions of input features

**Fig. 4:**
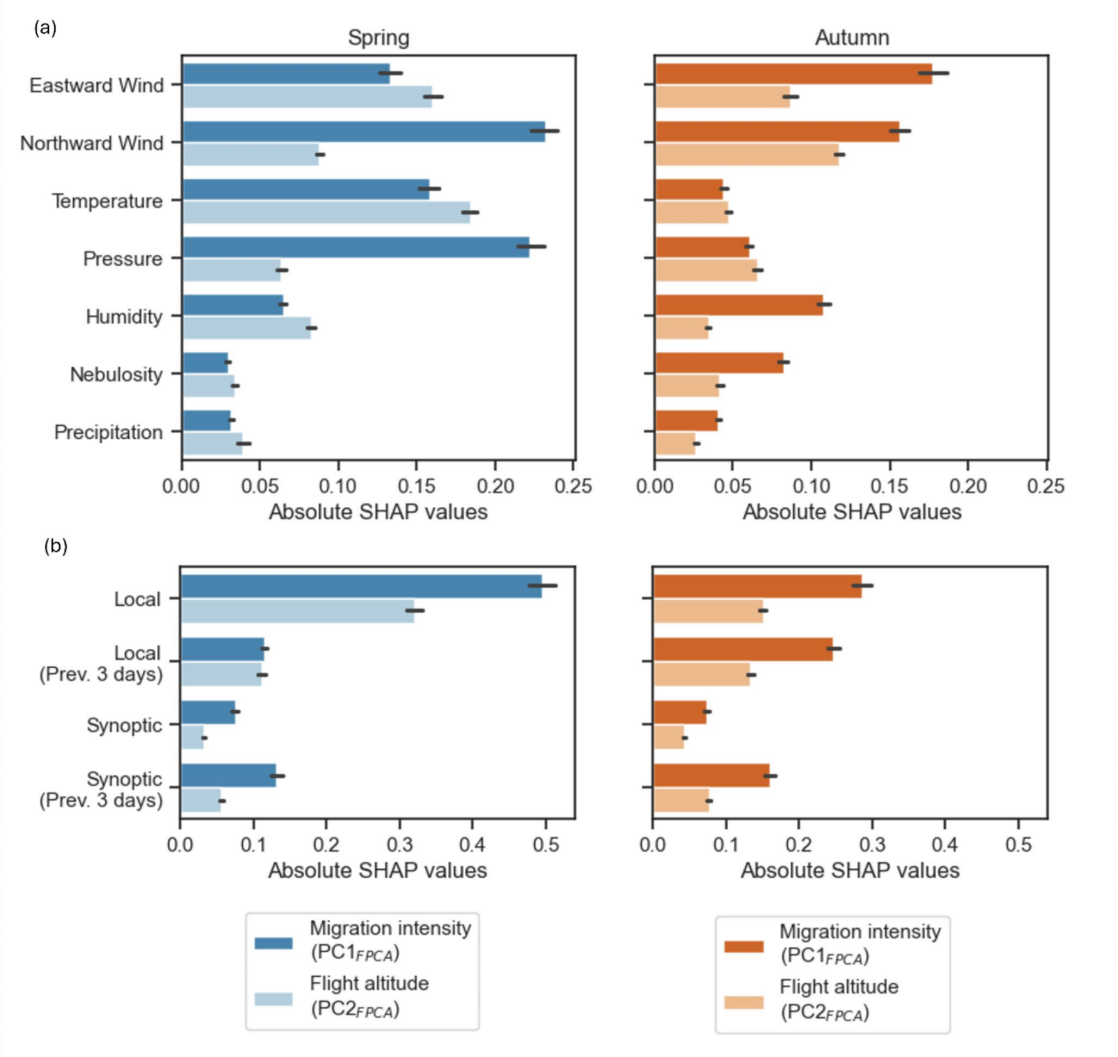
(**a**) Contributions of explanatory covariates aggregated by weather metrics based on the absolute SHAP values. (**b**) Contributions of explanatory covariates aggregated by spatio-temporal scales. These values are estimated separately for spring and autumn migration (left and right panels) and for the two main components of bird density profiles (PC1_FPCA_ and PC2_FPCA_)

The zonal and meridional components of wind had the most significant impact on predicting migration intensity (PC1_FPCA_) and flight altitude (PC2_FPCA_) across both seasons (Fig. 4). In spring, temperature and pressure were particularly influential, while in autumn, humidity and cloudiness had higher contributions. Local and instantaneous weather data had the highest contribution overall. However, the contribution of local data from previous days was notably higher in autumn compared to spring. The use of synoptic data contributed equally to both spring and autumn, especially when considering data from the previous three days. More specifically, temporal data played a crucial role in detecting migration peaks (Fig. 3). In contrast, local and instantaneous data were primarily essential for identifying nights with unfavorable conditions and low migration fluxes (Fig. 3).

**Fig. 5:**
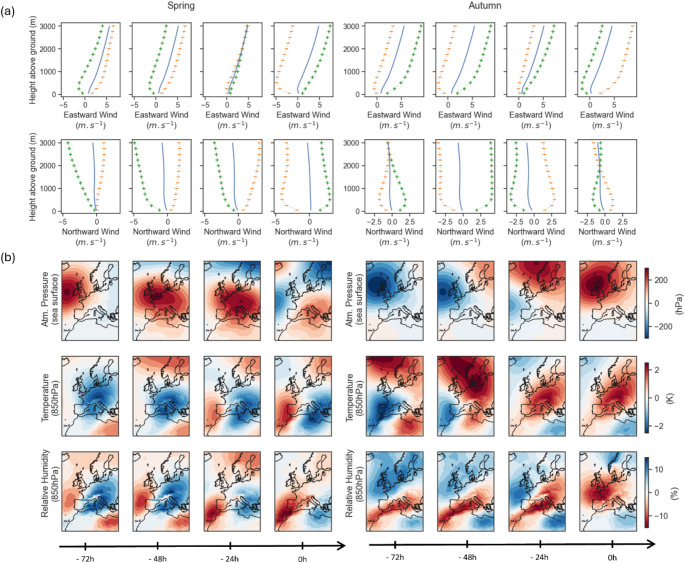
(**a**) Favorable local wind profiles reconstructed from averaged PC_FPCA_ overall (blue), associated with positive (green +) and negative SHAP values (orange -). (**b**) Favorable synoptic weather maps for bird migration through France reconstructed from averaged PC_EOF_ when SHAP values were positive. Each column is associated with a specific time before a migration night as detailed by the timeline at the bottom of the figure. Left and right panels refer to respectively spring and autumn migrations. The maps extend from − 20°W to 10°W longitude and 10°N to 70°N latitude

For both seasons, most favorable conditions were associated with low supportive winds preceded by 1–3 days of strong headwind (Fig. 5). Altitudes for strongest supportive winds were clearly higher in spring than autumn based on wind vertical profiles. In spring, these favorable conditions were associated with a high-pressure system over the Mediterranean Sea causing low South-West winds over the French territory. These conditions were following the arrival of relatively warm and dry air masses on the Iberian Peninsula and over the Strait of Gibraltar (Fig. 5). In autumn, the most favorable migration nights were associated with a strong high-pressure over the UK associated with North-East winds in France. This situation was also correlated with relatively warm and dry conditions across Europe and particularly above the England channel, the North Sea, and the continental Eastern Europe (Fig. 5). This was however preceded by a strong westward wind flux characterized by a low-pressure system bringing high relative humidity over the UK and Northern Europe (Fig. 5).

## Discussion

### Phenology trend

The phenology trend described in (Fig. 1) was consistent with the actual knowledge of bird migration in France and results from recent papers (Nilsson et al. 2019; Nussbaumer et al. 2021a; Weisshaupt et al. 2018). As illustrated by the PC1_FPCA_, the most important nocturnal migration intensities were recorded in the central part of France, from the Belgian border to the Spanish coast. Results from PC2_FPCA_ also follow the actual knowledge of flight altitudes of nocturnal migrating birds, with birds usually flying at higher altitudes in spring than in autumn due to favorable following southwesterly winds in spring (Bruderer et al. 2018). Flying altitudes are also expected to be higher at locations close to mountains. The pattern observed at Toulouse, located between the Pyrenees and the Massif Central, is in line with this expectation.

### Contributions of weather patterns to bird migration forecast

#### Local and instantaneous weather

In line with the literature, we found a strong relationship between bird migration fluxes and local and instantaneous weather conditions. Favorable conditions are indeed mostly characterized by supportive winds for both seasons (Liechti 2006; Nussbaumer et al. 2022). This is also reflected at the synoptic scale by our results, where bird fluxes occurred at the western and eastern parts of high-pressure systems for spring and autumn migration respectively(Manola et al. 2020; Richardson 1990); Fig. 5). The contribution of other weather metrics was season-dependent but consistent with previous observations (Kranstauber et al. 2022; Van Doren and Horton 2018). Spring migration is better forecasted using temperature and pressure metrics, describing global mass air characteristics. In contrast, autumn migration depended more on precipitation-related metrics (cloudiness and humidity). It is important to note that the contributions of accumulated precipitations might be underestimated due to the bias from the inherent properties of weather radar-based bird density estimations, as most of the observations occurring during rain were removed. By eliminating all rain-contaminated profiles, it is indeed not possible to properly assess the relationship between rainfall and bird migration using statistical tools. Local and instantaneous data were the most contributive features, yet they mainly helped forecasting nights with no or few migrations and had limited contributions for the forecast of migration peaks (Fig. 3).

#### Temporal weather dynamics

We highlighted the importance of considering temporal weather patterns in predicting migration fluxes, notably for the identification of peaks. Previous studies have accounted for local weather changes and have typically illustrated their importance for increased forecast accuracy, notably in autumn (Erni et al. 2002; Kranstauber et al. 2022; Tschanz et al. 2020; Van Doren and Horton 2018). Taking into account such information makes it possible to forecast situations where birds accumulate at stop-over sites due to unfavourable weather conditions (Kranstauber et al. 2022), or when bird migration intensity decreases after several consecutive days of favourable weather conditions (Nilsson et al. 2019). Our results consistently highlight a stronger contribution of the local weather conditions of previous days in autumn compared to spring. This suggests that the ‘sit-and-wait’ hypothesis of optimal migration, where birds are supposed to benefit from waiting for favorable conditions, might hold better for autumn migration (Haest et al. 2019; Nilsson et al. 2013). However, we also demonstrate that synoptic weather dynamics are needed for forecasting migration in both autumn and spring, specifically for migration peaks. We were able to clearly identify which large-scale synoptic weather patterns were associated with these peak events. For instance, in spring, the highest migration was presumably associated with bird accumulation on the North-African coast and over the Gibraltar Strait due to North wind fluxes bringing cold and humid air to the Maghreb coasts (Fig. 5). This is in line with recent results that similarly identified these areas as important to predict migration timing at remote locations (Haest et al. 2020). Similarly, bird accumulation seems to occur in the UK and Scandinavia in autumn, as the highest predicted migration nights were following three consecutive days of rainy weather in these areas. This confirms previous studies expecting the North Sea, the English Channel and the Mediterranean Sea to function as ecological barriers, leading to birds accumulating en route due to unfavorable conditions (Bradarić et al. 2020; Clipp et al. 2020; Manola et al. 2020). These results provide thus promising areas for improvement, and to better understand, quantify and forecast the accumulation of birds at these specific geographical barriers, building on a multiscale consideration of weather phenomena (Shamoun-Baranes et al. 2017).

### Methodological considerations

#### Phenology trend and model evaluation

Interestingly, the use of a phenological trend as forecast model led to relatively high forecast accuracy. It even obtained better predictive results than the Van Doren and Horton (2018) approach in some cases (Table 1). For this reason, we formulated our problem as a standardized residual model. The main reason was to model the motivation of departing for migration rather than modelling the seasonal trend of bird migration, as proposed by Kranstauber et al. (2022). Using de-trending highlights therefore more clearly the contributions of different data inputs to intra-seasonal bird fluxes (Iler et al. 2017). We therefore encourage future publications to assess the model performance of bird migration forecast models on a detrended metric.

#### Dimension reduction tools

Our approach to nocturnal bird migration forecasting was characterized by several key methodological contributions including the use of dimension reduction tools, such as FPCA and EOF. FPCA described the vertical autocorrelation of bird density and local weather metric profiles. This is particularly relevant for improving bird migration forecasts in spring when birds tend to fly higher to reach supportive winds. In contrast, Van Doren and Horton (2018) only included weather information at a fixed altitude, which makes impossible to describe bird altitude adaptation related to wind preference during flights. We also accounted for synoptic weather patterns using EOF. To our knowledge, few studies use synoptic information in forecast models (Bradarić et al. 2024; Kranstauber et al. 2022). It is worth to note that PC_EOF_ have also indirectly been considered in previous bird migration studies through oscillation indexes such as the North Atlantic Oscillation index (Haest et al. 2018; Hüppop et al., 2003). EOF eliminates the need for expert-defined identification of description metrics and remote areas of interest, thereby increasing the transferability and applicability of our frameworks. Finally, these dimension reduction tools reduced the number of input features, optimizing computational resources.

#### Explainable trees & SHAP values

Regression trees have been extensively used in ecology for last twenty years (Breiman 2001). Many studies, however, consider these approaches to be limited for interpretation in comparison to usual generalized linear models. Thanks to recent tools such as SHAP (Lundberg et al. 2020), we have illustrated in this paper how it is now possible to explore and understand ecological processes from these complex regression trees. The combined use of dimension reduction tool and explainable regression trees enabled the identification of complex relationships between bird migration intensity and weather patterns, without a priori knowledge. This framework could therefore be used and transferred to other complex ecological systems.

### Limitations & perspectives

Despite significant improvements in identifying dates with strong passages of migratory birds, our approach remains limited in estimating the quantity of birds associated with these events (Fig. 3). The prediction of migration peaks is yet of critical importance for successful avifauna conservation efforts (Elmore et al. 2021; Horton et al. 2021). To accurately predict bird fluxes during high migration passage, we might need additional information on species’ migration phenology and population. Practically, a first model could estimate the number of birds ready to depart based on their phenology and distributions, and a second model could forecast the proportion of birds flying based on weather conditions. This approach was initially proposed by Kranstauber et al. (2022). In our work, we followed the same principle by separately modelling phenology on one side and training a residual model on the other.

#### Modelling bird phenology

Future improvements could significantly improve the phenological model by including complementary data describing migration patterns (e.g. citizen science, acoustic data, ringing data and bird radars) (Van Doren et al. 2023; Weisshaupt et al. 2021). Weather radars provide only vertical information and are not able to get insights on bird fluxes at the population level. Citizen science and ringing data could help identifying some specific migration phenology patterns at a population level. Bird radar data could also lead to increased spatial resolution of these patterns and their relation to orography, land types and other geographical features (Bruderer et al. 2018; Bruderer and Peter 2022; Kranstauber et al. 2022).

#### Forecasting bird migration peaks in response to weather conditions

Weather radars observe a volume of different species and populations which react to weather in different ways, and which have different migration phenology. For that reason, forecasting migration peaks derived from weather radar is challenging. The addition of information at species and population level could be expected to make a significant improvement. In addition, the difficulty of forecasting migration peaks may also be related to the size of the datasets. Migration peaks are infrequently represented in the available data, and some studies suggest that more data may be necessary for improved predictions (Kranstauber et al. 2022). In particular, the impact of extreme weather situations on migration can be particularly difficult to assess using statistical tools based on small datasets. Gradient-boosted trees might also be limited for capturing complex spatial and temporal dynamics of local and synoptic weather patterns, in comparison with recent predictive tools such as deep neural networks (Lam et al. 2023; Ravuri et al. 2021). An alternative could also be to predict migration peaks without necessarily seeking to estimate the associated bird density (van Gasteren et al. 2019). By delving deeper into the relationships between synoptic weather patterns over the preceding days and bird migration peaks, we anticipate substantial improvements in the performance and relevance of operational bird migration forecasts.

## Electronic supplementary material

Below is the link to the electronic supplementary material.

Supplementary Material 1

## Data Availability

We plan to share data/code on a public repository.
