# Supplementary material for: Enhanced forecasting of bird nocturnal migration intensity in relation to previous days and synoptic weather patterns
Source: Int J Biometeorol. 2025 Apr 19;69(7):1617–30. doi: 10.1007/s00484-025-02917-4 (PMC12178967; doi:10.1007/s00484-025-02917-4)
Supplement: Supplementary file 1 — Supplementary Material 1 [file 484_2025_2917_MOESM1_ESM.docx]

Enhanced forecasting of bird nocturnal migration intensity in relation to previous days and synoptic weather patterns

Amédée Roy^1,*^, Thibault Désert^2^, Vincent Delcourt^3^, Cécile Bon^1^ & Baptiste Schmid^4^

^1^ France Energies Marines, 525 Avenue Alexis de Rochon, 29280 Plouzané, France

^2^ Météo-France, 42 Avenue Gaspard Coriolis, 31100 Toulouse, France

^3^ Biotope, 22 Boulevard Maréchal Foch, 34140 Mèze, France

^4^ Swiss Ornithological Institute, Seerose, 1, Sempach, CH-6204, Switzerland

*Corresponding author

**Supporting information**

### Radars characteristics

| Abbreviation | Name | Band Type | Longitude (°) | Latitude (°) | Antenna height above sea level (m) |
| --- | --- | --- | --- | --- | --- |
| ABBE | Abbeville | C | 1.83472 | 50.13582 | 83.6 |
| BORD | Bordeaux | C | -0.69188 | 44.83147 | 70 |
| BOUR | Bourges | C | 2.35944 | 47.0586 | 173.5 |
| CAEN | Falaise | C | -0.14944 | 48.92721 | 167.4 |
| NANC | Nancy | C | 6.58333 | 48.71667 | 296.3 |
| NIME | Nimes | S | 4.50269 | 43.80614 | 78.1 |
| NIZI | St Nizier | C | 4.44555 | 46.06639 | 919.8 |
| TOUL | Toulouse | C | 1.3763 | 43.57432 | 187.1 |
| TREI | Treillieres | C | -1.65389 | 47.33555 | 81 |

### Hyperparameter space for gradient-boosted trees optimization

### The hyperparameters of gradient-boosted trees have been optimally selected among the following values.

| Parameters | Description | Values |
| --- | --- | --- |
| loss | Loss function | choice(squared_error, absolute_error) |
| learning_rate | Learning rate | interval(0.001, 0.1) |
| max_iter | Maximum number of trees | range(100,1000,step=100) |
| l2_regularization | L2 regularization parameter | interval(0, 1) |
| max_leaf_nodes | Maximum number of leaves for each tree. | choice(4,8,16,32,64) |
| min_samples_leaf | The minimum number of samples per leaf | choice(4,8,16,32,64) |

### Insect and Birds filter moments


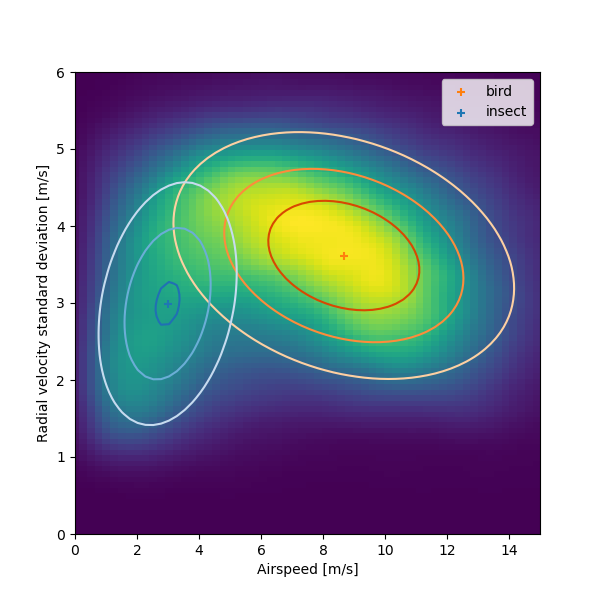
Following Nussbaumer et al. (2021), we implemented a mixture of Gaussian distribution in order to distinguish bird-like from insect-like densities estimated from the vol2bird algorithm. We obtained very similar results which support the relevance of their approach. For more information, please refer to Nussbaumer et al. (2021).

Illustration of the 2D Gaussian Mixture Model as proposed by Nussbaumer et al., 2021 to differentiate insect-like from bird-like densities estimated from vol2bird
